# Delphi consensus on the real-world application of the updated diagnostic and treatment guidelines for migraine in Asia Pacific

**DOI:** 10.3389/fneur.2026.1770966

**Published:** 2026-05-28

**Authors:** Charles Hua Chiang Siow, Shengyuan Yu, Surat Tanprawate, Shuu-Jiun Wang, Elspeth J. Hutton, Byung-Kun Kim, Min Kyung Chu, Norihiro Suzuki, Wanakorn Rattanawong, Ka Sing Wong, Mi Ji Lee, Zhao Dong, Hebo Wang, Xianliang Li, Koichi Hirata, Takao Takeshima

**Affiliations:** 1 The Brain Centre, Mt Elizabeth Novena Hospital, Singapore, Singapore; 2 Department of Neurology, Chinese PLA General Hospital, Beijing, China; 3 Department of Internal Medicine (Neurology Division), Faculty of Medicine, Chiang Mai University, Chiang Mai, Thailand; 4 Neurological Institute, Taipei Veterans General Hospital, Taipei, Taiwan; 5 College of Medicine and Brain Research Center, National Yang Ming Chiao Tung University, Taipei, Taiwan; 6 Department of Neuroscience, School of Translational Medicine, Monash University, Melborne, VIC, Australia; 7 Department of Neurology, Alfred Health, Melbourne, VIC, Australia; 8 Department of Neurology, Eulji University School of Medicine, Daejeon, Republic of Korea; 9 Department of Neurology, Yonsei University College of Medicine, Seoul, Republic of Korea; 10 Shonan Keiiku Hospital, Fujisawa, Japan; 11 Faculty of Medicine, King Mongkut's Institute of Technology Ladkrabang, Bangkok, Thailand; 12 Division of Neurology, Department of Medicine and Therapeutics, The Chinese University of Hong Kong, Hong Kong, China; 13 Department of Neurology, Seoul National University Hospital, Seoul National University College of Medicine, Seoul, Republic of Korea; 14 Department of Neurology, Hebei General Hospital, Shijiazhuang, China; 15 Department of Neurology, The Second Affiliated Hospital of Guangzhou Medical University, Guangzhou, China; 16 Department of Neurology, Dokkyo Medical University, Mibu, Tochigi, Japan; 17 Tominaga Hospital, Osaka, Japan

**Keywords:** anti-CGRP mAbs, APAC, CGRP mAbs, delphi, gepants, ICHD-3, migraine diagnosis, migraine prevention

## Abstract

**Background:**

Although diagnostic criteria and treatment guidelines for migraine are well-established, care remains suboptimal due to frequent misdiagnosis and undertreatment. This Delphi consensus aimed to explore how clinicians in the Asia-Pacific region approach migraine diagnosis and management.

**Methods:**

This study used a modified-Delphi method, involving two rounds of surveys and a virtual scientific meeting with 14 headache-specialists from seven countries in Asia-Pacific. Consensus on statements was measured using a Likert scale ranging from 1 to 9, with consensus threshold set at 75%.

**Results:**

The study had 100% participation in both surveys, and the scientific meeting was attended by 64.3% of participants. Out of 21 closed-ended statements, 12 reached consensus. Notably, 93% of participants reported using only ICHD-3 for migraine diagnosis, and 79% found the criteria easy to apply. For the diagnosis of migraine, all participants evaluated migraine-related disability and depression; 93% anxiety, and 86% sleep disturbances. Neck pain and dizziness were recognised as migraine-associated symptoms by 93% of respondents, with 85% reporting that 25–75% of their patients experience neck pain. Anti-CGRP mAbs were considered effective and addressed unmet needs for chronic or episodic migraine by 86% of participants. Efficacy (100%) was the primary deciding factor in selecting a specific anti-CGRP mAb, followed by safety (93%) and tolerability (86%). Factors such as frequency of administration (43%), perceived wearing-off effect (43%), availability and reimbursement (57%), and cost (58%) were less influential in decision-making. While 79% of the participants would consider anti-CGRP mAbs as first-line treatment, 93% believed these therapies would improve safety, adherence, and compliance outcomes. Only 7% of the participants favoured the use of gepants. Participants unanimously supported the use of anti-CGRP mAbs in adolescents (13–17 years), but not in children (6–12 years).

**Conclusion:**

Consensus was reached on key aspects of migraine diagnosis and management. Participants endorsed the ICHD-3 criteria as the diagnostic standard and favoured using headache diaries, followed by MIDAS and HIT-6 for assessment. Anti-CGRP mAbs were recognised as effective in addressing unmet needs in migraine prevention. The panel advised against using anti-CGRP mAbs in children under 12 due to insufficient evidence, highlighting the need for more paediatric data.

## Highlights

- This modified Delphi study was conducted with 14 headache specialists across Asia-Pacific.
- Consensus was reached on 12 of 21 statements on key aspects of migraine diagnosis and management.
- 93% used ICHD-3 for diagnosis; 79% found them easy to apply.
- 86% considered anti-CGRP mAbs effective.
- Efficacy (100%), safety (93%), and tolerability (86%) are top selection criteria, followed by cost, availability, and administration frequency.

## Introduction

Migraine is a chronic neurological condition characterised by recurrent episodes of unilateral, pulsating headaches that worsen with physical activity and are accompanied by symptoms such as photophobia, phonophobia, nausea, and vomiting (1). It is a common headache disorder affecting approximately 15% of the adult population and is ranked as the second most disabling disease globally (2). Approximately 4.9 million Australians, or 20.5% of the population, are affected by the disease (3). In Thailand, the prevalence is higher, impacting around 29% of the population (4). Across Asia, migraine affects an estimated 6 to 14% of the general population (5, 6). A study by Takeshima et al. covering China, Japan, and South Korea reported a 1-year migraine prevalence (based on International Headache Society (ICHD) criteria) ranging from 6.0 to 14.3% among adults with the highest rates observed in females aged 30–49 years (11–20%) and males in the same age group (3–8%). The study revealed substantial gaps in diagnosis and care: over half of the Chinese participants had previously consulted a physician, yet around 50% of those with headaches had never received a formal migraine diagnosis, and only 13 to 18% had a documented history of migraine. In Japan, a significant proportion (59–72%) had never sought medical attention, and only 12% were aware of their condition, while in South Korea the 1-year prevalence was 5.2 to 6%, with physician consultation rates between 31 and 34%. Together with other Japanese data showing similarly low rate healthcare-seeking behaviour, these findings highlight the significant unmet needs in migraine across the Asia-Pacific (APAC) region, particularly in terms of accurate diagnosis and effective management of migraine (5, 7). Furthermore, migraine carries a significant financial burden, with annual total costs in the USA estimated at 27 billion USD (8). In addition to the financial burden, migraine is also associated with work absenteeism and reduced productivity among adults during their important working years (9).

Accurate diagnosis of migraine remains challenging due to the heterogeneous presentation of the symptoms, inherent subjectivity in perception of pain intensity, and an overlap in symptoms between various types of headaches. These factors often contribute to misdiagnosis or delayed treatment (10). Therefore, to enhance diagnostic accuracy, it is imperative that the clinician utilise predefined criteria and tools, in addition to clinical judgement and experience. The International Classification of Headache Disorders, 3rd edition (ICHD-3) provides standardised criteria and guidelines to assist healthcare professionals in the accurate diagnosis and classification of headache disorders. According to these guidelines, the diagnosis of migraine is based on five key criteria: frequency: at least five attacks; pain characteristics: typically unilateral, pulsating, of moderate to severe intensity, and worsened by routine physical activity; duration: lasting between 4 and 72 h; associated symptoms: such as nausea and/or vomiting, photophobia, and phonophobia; exclusion: other potential causes must be ruled out (1). Understanding how to effectively implement these guidelines in diverse clinical settings is critical to improving diagnostic accuracy and optimising patient outcomes. In fact, a multi-national study showed the most common diagnosis was migraine for patients with headache as the chief complaint in neurological service in Asia (11).

The primary goal of migraine treatment is to relieve symptoms and prevent future attacks. Migraine treatments consist of both acute/abortive medications and preventive treatments alongside various non-pharmacological options. Acute medications include paracetamol, non-steroidal anti-inflammatory drugs (NSAIDs), and triptans, while the use of ergot alkaloids and adjunct antiemetics is less common (12). Preventive medications aim to reduce the frequency, severity, or duration of migraine attacks in individuals for whom acute treatments alone are insufficient. These typically include beta-blockers, tricyclic antidepressants, serotonin-norepinephrine reuptake inhibitors, anti-epileptic medications, anti-calcitonin gene-related peptide (CGRP) monoclonal antibodies (mAbs; mAbs that target CGRP or its receptor), and gepants, among others (13, 14).

In recent years, the progress of migraine-related research, especially the discovery of new therapeutic targets such as CGRP, has led to important advances in the treatment of migraine. Currently, there are four mAbs available acting on the CGRP pathway, which can be used for migraine prevention: three targeting the CGRP ligand (fremanezumab, eptinezumab, and galcanezumab); one targeting the CGRP receptor (erenumab).

The consensus statement by the European Headache Federation (EHF) recommends the utilisation of anti-CGRP mAbs as a first-line treatment option in individuals with migraine who require preventive treatment (15). Despite the availability of treatment options and established diagnostic criteria, clinical care for migraine remains insufficient, with misdiagnosis and undertreatment representing significant public health concerns. A comprehensive approach is required to promote accurate diagnosis and the implementation of evidence-based management strategies. We therefore conducted a Delphi consensus to understand Headache specialists’ approaches in diagnosing and treating patients with migraine in APAC.

## Methods

### Study design

This study used a modified Delphi method, involving two rounds of cross-sectional surveys and a virtual scientific meeting with 14 headache specialists from seven countries. Cross-sectional surveys were conducted between August and November 2024, while a scientific meeting was conducted in September 2024. A research team developed a survey questionnaire, which was administered by an independent vendor using Decipher software (version Compact = 153). Survey responses were analysed after each round using Microsoft Excel, with all participant data handled with strict confidentiality and anonymity. Since this was a non-interventional physician survey, no formal ethical approval was required.

### Participants

This study included 14 headache specialists from seven countries (Australia, Mainland China, Hong Kong, Japan, South Korea, Taiwan, and Thailand). Additionally, expert chairperson and co-chair from Singapore and China, respectively, were invited to help identify qualifying experts, moderate the scientific meeting, and provide independent feedback throughout the Delphi process. Participants were carefully selected utilising the following criteria:

a Experience

- Headache specialist with a minimum of 5 years of independent clinical experience in treating migraine patients

b Specialisation

- Predominantly neurologists and dedicated headache specialists, with some participants also practising in general neurology or pain clinics

c Geographic distribution

- Experts from multiple East and South-East Asian countries as well as Australia to avoid dominance by any single health-care system

d Employment

- Department head or full-time professor at a university in a relevant therapeutic area
- Full-time physician at a leading national clinical centre

e Publications

- Author or editor of a leading textbook
- Published frequently in leading journals of a relevant therapeutic area

f Speaking

- Serve as a speaker or chair sessions at national or international conferences

g Associations

- Serve in a leadership role of an international or national medical association, member of a scientific academy

All experts initially invited to the panel were included throughout the entire Delphi process. Attendance at the scientific meeting was not mandatory, and absence from the meeting did not affect eligibility for participation in subsequent survey-2, which involved reviewing and responding to the consensus statements refined during the scientific meeting.

### Study stages

#### Development of the survey questionnaire

The first survey was developed using key statements derived from a targeted literature review focused on the objectives of this Delphi consensus.

A comprehensive literature search was conducted via the PubMed database utilising different objective-specific search terms. Additional searches were conducted in Google Scholar and from other review article reference lists through cross-referenced articles. The search was not limited by time, and therefore, all applicable literature was screened. Only studies conducted in human populations and published in the English language were considered, and all the retrieved articles were screened for population and objectives.

Based on this literature review, the research team developed an initial pool of candidate statements, which were iteratively refined through virtual discussions with the expert chairperson and co-chair to ensure clinical relevance, clarity, and applicability across Asia-Pacific practice settings. The draft statements and response options were subsequently reviewed and pilot tested to assess face and content validity. Minor wording revisions were made by consensus before finalising survey-1.

The questionnaire consisted of 21 Likert-style statements, 17 open-ended questions, 17 yes/no type questions, 6 radio select questions, 2 ranking questions (*n* = 63), and was developed to address the following themes:

- Current clinical diagnosis of migraine
- Updated treatment concepts with anti-CGRP mAbs in migraine preventive management.

#### Survey-1 and scientific meeting

The first round of the Delphi survey was distributed via email and completed online in August 2024. Participants from the first round of the survey were subsequently invited to a virtual meeting in September 2024 to review the survey findings and provide expert input, with particular emphasis on statements that had not reached consensus in Survey-1. This scientific meeting was facilitated by the co-chairs. The sponsor did not participate in the survey process or the meeting discussions.

#### Survey-2 and final data analysis

Statements which did not reach consensus in survey-1 and any additional statements deemed appropriate from the scientific meeting were included in survey-2 (1 Likert-style, 1 yes/no, 2 radio-select, and 1 ranking). Survey-2 was e-mailed to the participants and was completed in October/November 2024.

### Data analysis

Only fully completed questionnaires were taken into consideration for descriptive analysis. A 1-9-point Likert scale was used to phrase the statements and rate responses as per best practice. Participants rated their level of agreement with each statement anonymously, ranging from 1 (strongly disagree) to 9 (strongly agree). These scores were divided into three groups: agree (7–9 points), neither agree nor disagree (4–6 points), and disagree (1–3 points). Open-ended, ranking on preference statements, Yes/No statements, and single/multiple-select radio button selection statements were quantified. For these quantitative items, frequencies and percentages were calculated for each response category. Ranking items were summarised by the proportion of responses at each rank, with weighted scores computed to derive overall item rankings. Yes/no and single- or multiple-select questions were reported as the proportion of participants selecting each option. Free-text responses from each survey round were thematically grouped into broad categories and reported as the proportion of participants contributing to each category. Consensus was deemed to have been reached in both rounds of the survey when ≥75% of the respondents scored a characteristic within the same range. This consensus threshold of 75% was consistent with previous studies that have employed the Delphi method (16, 17).

## Results

All the invited participants completed both surveys, resulting in a 100% response rate. The scientific meeting was attended by 9 of 14 participants (64.3%). Overall, consensus was reached for 12 out of 21 closed-ended statements.

### Current clinical diagnosis of migraine

**Figure 1:**
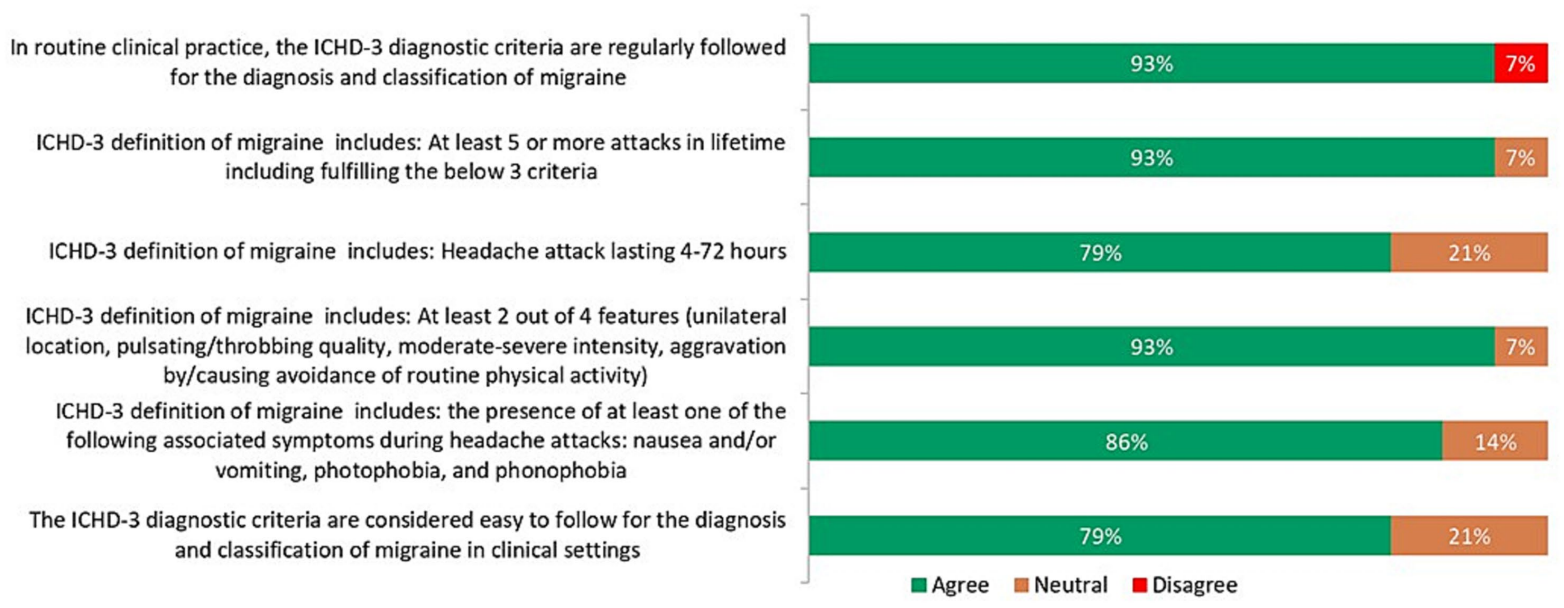
Consensus statements: current clinical diagnosis of migraine. ICHD-3, International Classification of Headache Disorders, third edition.

Figure 1 summarises all the consensus statements related to the current practises for clinical diagnosis of migraine. Further quantitative data of all the statements is summarised in the Supplementary Table S1.

Among the 14 participants, 93% reported using the ICHD-3 criteria for diagnosing and classifying migraine in routine clinical practice, reaching consensus. Out of 14 participants, 13 confirmed that they do not use any additional diagnostic criteria. The one participant who did, referred the “Guidelines for Diagnosis and Treatment of Migraine in China,” which aligns with ICHD-3.

**Figure 2:**
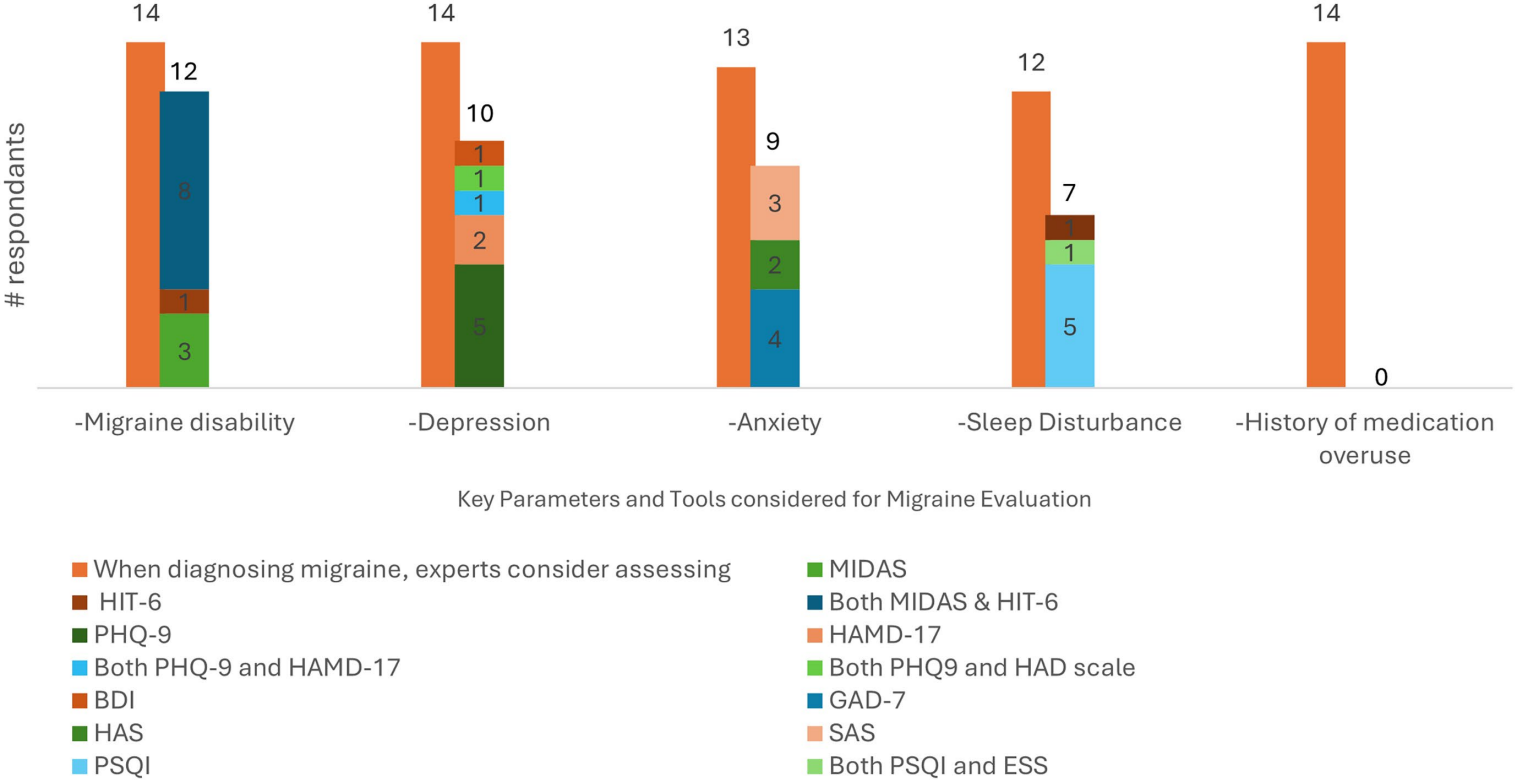
Key parameters and tools considered for migraine evaluation. BDI, Beck Depression Inventory; ESS, Epworth Sleepiness Scale; GAD-7, Generalised Anxiety Disorder 7-item scale; HAMD-17, Hamilton Depression Rating Scale (17 items); HAD, Hospital Anxiety and Depression Scale; HAS, Hamilton Anxiety Scale; HIT-6, Headache Impact Test (6 items); MIDAS, Migraine Disability Assessment Scale; PHQ-9, Patient Health Questionnaire-9; PSQI, Pittsburgh Sleep Quality Index; SAS, Self-Rating Anxiety Scale.

Participants also agreed with specific components of the ICHD-3 definition: 93% agreed on the requirement of at least five lifetime attacks, 79% agreed on attack duration of 4–72 h, 93% agreed on having at least two of the four pain features (unilateral location, pulsating quality, moderate/severe intensity, and aggravation by routine activity), and 86% agreed on the presence of at least one associated symptom (nausea/vomiting, photophobia, phonophobia). Additionally, 79% found the ICHD-3 criteria easy to apply in clinical practice. In routine clinical practice when diagnosing migraine, all participants reported assessing migraine disability, depression and patients’ history of medication overuse, while majority (93%) also evaluated anxiety and sleep disturbances. Around two-thirds of participants (12) reported using at least one validated instrument to assess migraine disability; most commonly the Migraine Disability Assessment Scale (MIDAS), and/or Headache Impact Test-6 (HIT-6), while a few relied on simple measures such as clinical evaluation or headache frequency. For depression, anxiety, and sleep disturbances, most participants reported using validated assessment tools (10, 9, and 7 participants respectively), while very few relied on simpler approaches such as clinical interviews, clinical history, or routine clinical evaluation (Figure 2). Use of specific instruments and respective details for depression, anxiety, and sleep disturbance is detailed in Supplementary Table S1.

For evaluating patients with chronic or episodic migraine, participants ranked headache diaries capturing monthly migraine days (MMDs) as the most validated diagnostic assessment/screening tool followed by quality-of-life assessments, with MIDAS ranked second, HIT-6 third, the Migraine-Specific Quality of Life Questionnaire (MSQ) fourth, and the 24-h Migraine Quality of Life Scale (MQoLQ) fifth (Supplementary Table S1).

**Figure 3:**
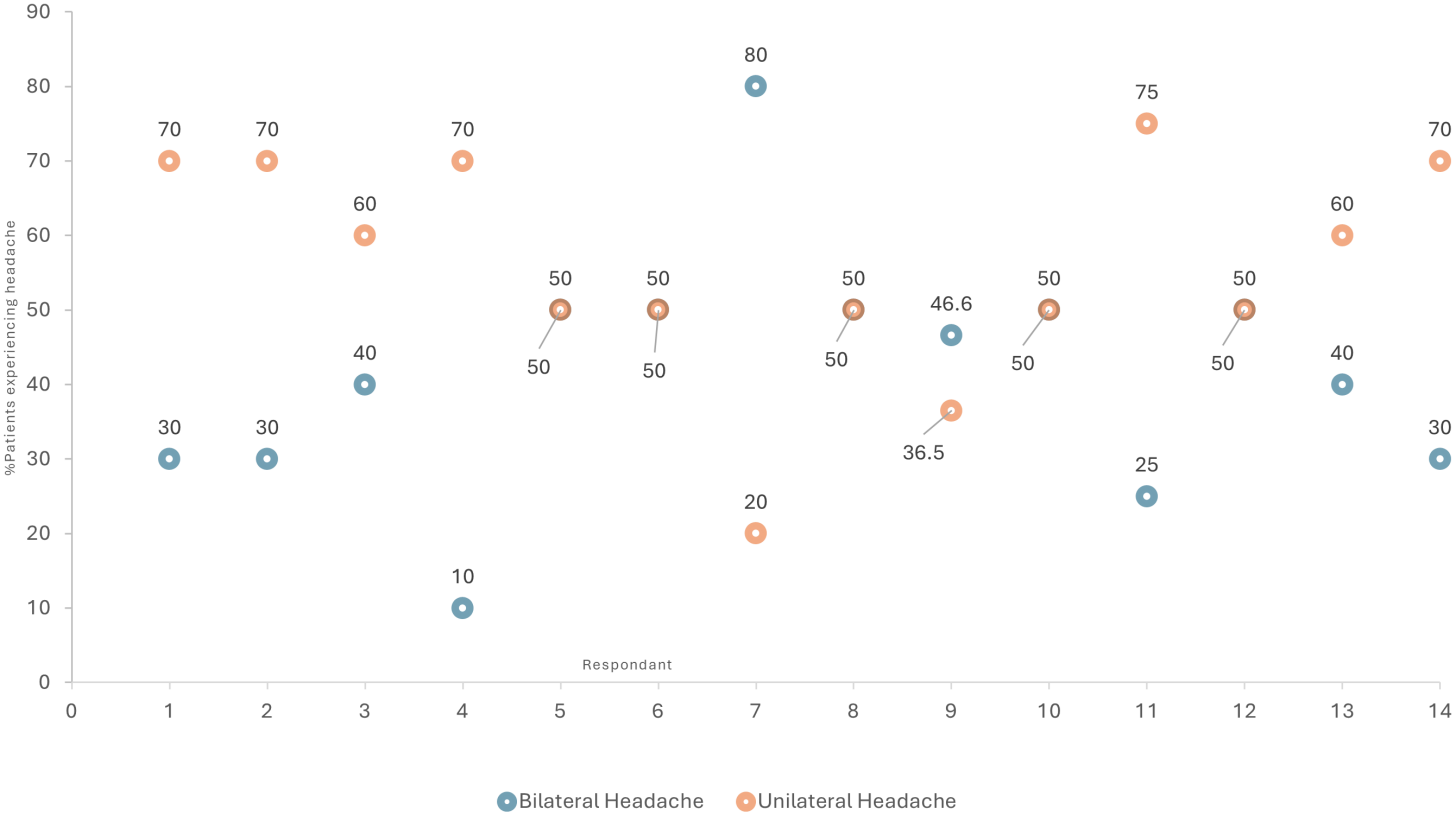
Reported headache patterns in migraine: unilateral vs. bilateral headache.

Participants reported that, in their clinical experience, unilateral headache was more common than bilateral headache in patients with migraine, accounting for approximately 57 and 43% of headache presentations, respectively (Figure 3; Supplementary Table S1).

Neck pain and dizziness were considered to be associated symptoms of migraine by 93% of participants, with 85% of participants noting that 25 to 75% of their patients exhibit neck pain. Among the 14 participants, 46% reported that dizziness affects 25 to 75% of their patients, while the remaining 54% indicated that dizziness occurs in ≤25% of their patients (Supplementary Table S1).

### Updated treatment concepts with anti-CGRP mAbs in migraine preventive management

**Figure 4:**
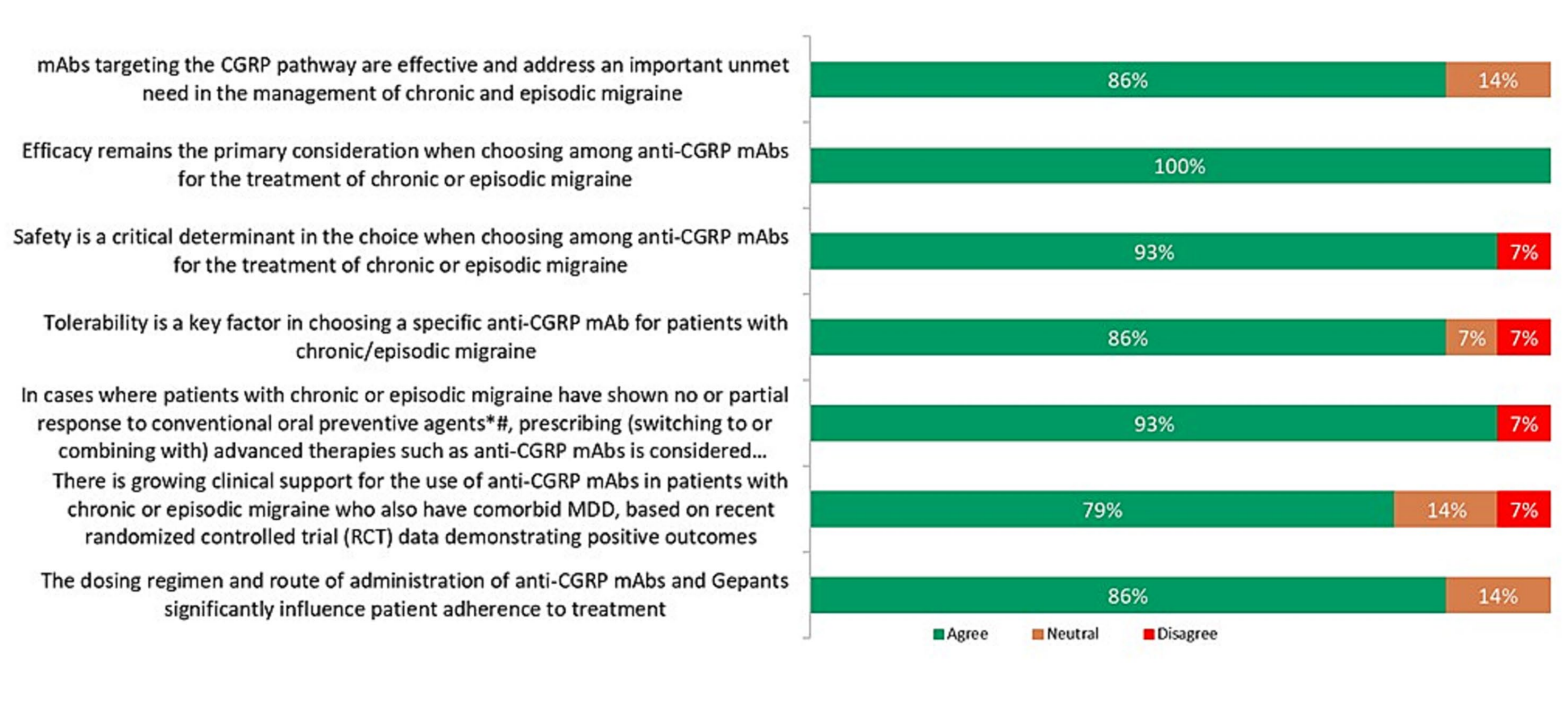
Consensus statements: updated treatment concepts with anti-CGRP mAbs in migraine preventive management. Anti-CGRP mAb, anti-Calcitonin Gene-Related Peptide monoclonal antibody; MDD, Major Depressive Disorder.

Figure 4 summarises all consensus statements related to the treatment with anti-CGRP mAbs. Further quantitative data of all statements is summarised in the Supplementary Table S2.

Majority of participants (86%) agreed that monoclonal antibodies (mAbs) targeting the CGRP pathway are effective and fulfill the unmet need in patients with chronic/episodic migraine, reaching consensus. All participants agreed that efficacy is the primary deciding factor in choosing a specific anti-CGRP mAb for patients with chronic/episodic migraine, with safety and tolerability also considered key determinants. In contrast, frequency of administration, wearing off effect, availability/reimbursement, and cost (if it is an out-of-pocket expenditure for the patient) were not considered as deciding factors and did not reach consensus. However, when reassessed in survey-2, the participants ranked cost to be the most important factor in addition to safety and efficacy, followed by availability/reimbursement (rank 2), frequency of administration (rank 3), and wearing off effect (rank 4; Supplementary Table S2).

If cost is not a concern, most of the participants (79%) indicated that they would consider anti-CGRP mAbs as first-line treatment, while very few (21%) indicated that they would consider them as second-line treatment (Supplementary Table S2).

For migraine patients showing a positive response to anti-CGRP mAbs in the follow-up visit/assessment, participants reported variability in considering continued treatment for 6 months (36%), 12 months (43%), and more than 12 months (21%; Supplementary Table S2).

When prescribing anti-CGRP mAbs, participants indicated no consensus in dosing regimen preference: 28% preferred monthly regimen, 36% quarterly regimen, and 36% recorded no preference. Additionally, patients’ preferences were always taken into consideration, as indicated by all participants (Supplementary Table S2).

Among participants who reported treating migraine in children/ adolescents (6–17 years old) in their routine clinical practice (10 out of 14), all indicated that they would consider anti-CGRP mAbs only for the 13–17 years age group and not for the 6–12 years age group (Supplementary Table S2).

In survey-1, consensus was not reached regarding combining with or switching to advanced therapies like anti-CGRP mAbs when patients with chronic/episodic migraine have failed (no response or partial response) while already taking other oral migraine preventive agents including topiramate, divalproex sodium/valproate sodium, beta-blocker: e.g. metoprolol, propranolol, timolol, atenolol, nadolol, tricyclic antidepressant: e.g. amitriptyline, nortriptyline and serotonin-norepinephrine reuptake inhibitor: e.g. venlafaxine, duloxetine. This disagreement was discussed during the scientific meeting, and participants concluded that the disagreement reflected differences in national guidelines for selecting advanced therapies in patients with failure to several oral preventive agents. Consequently, the question was rephrased in the second survey from a broader perspective, considering the failure of any oral migraine preventive agent. When reassessed after rephrasing in survey-2, the participants reached consensus (93% agreement) for prescribing (either combine with or switch to) advanced therapies like anti-CGRP mAbs when patients with chronic/episodic migraine have failed (no response or partial response) previous oral migraine preventive agents. Among these 13 experts who agreed, preferences regarding specific switching vs. combination strategies showed a varied opinion, reflecting differing clinical contexts; these detailed patterns are reported in Supplementary Table S2.

**Figure 5:**
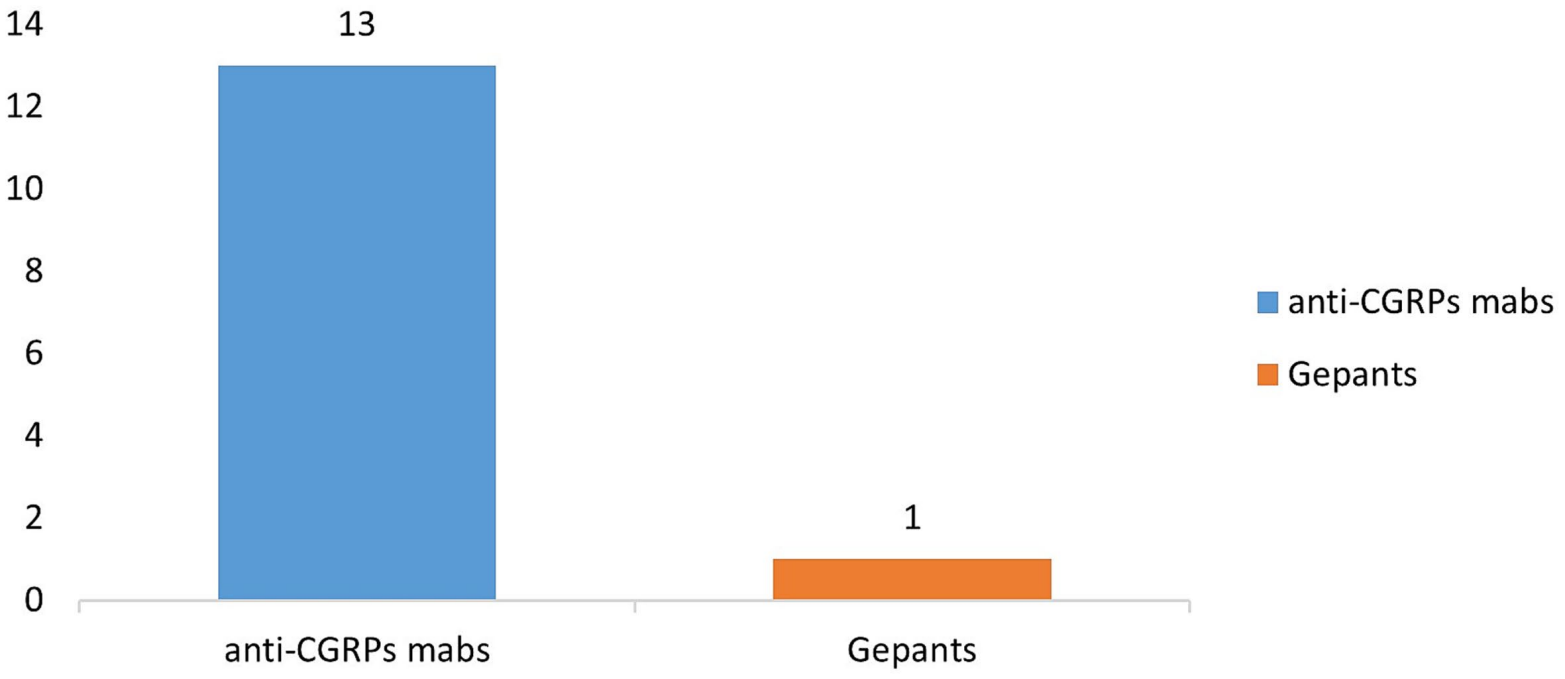
Anti-CGRP mAbs vs. gepants: clinician preferences for migraine treatment outcomes.

Majority of the participants (93%) indicated that anti-CGRP mAbs are likely to result in better clinical outcomes in terms of safety, adherence and compliance in patients with chronic/episodic migraine, while only 7% reported better clinical outcomes with gepants (Figure 5; Supplementary Table S2).

There was also consensus (86% agreement) that while prescribing anti-CGRP mAbs/gepants, dosing regimen and route of administration has an impact on patient’s adherence to the treatment.

The participants reached consensus (79% agreement) that anti-CGRP mAbs can be prescribed in patients with chronic/episodic migraine who have comorbid conditions, such as depression or major depressive disorder (MDD). Additionally, 70% reported that they either receive referrals from or refer such patients to psychiatrists for follow-up care.

## Discussion

The expert panel in this Delphi consensus demonstrated broad agreement on the key aspects of diagnosis and treatment of migraine, including the central role of ICHD-3 guidelines and the use of preventive agents for the management of chronic/episodic migraine. Overall, consensus was reached for more than half of the statements, particularly those relating to use of ICHD-3 criteria and the general role of preventive treatment. By contrast, statements on the precise positioning of anti-CGRP therapies and sequencing of preventive options did not reach consensus, likely reflecting differences in national policies, availability of specific agents, and local treatment pathways across participating countries.

ICHD-3 outlines operational diagnostic criteria, including well-defined rules on the feature combinations required to establish a diagnosis (18, 19). In this study, consensus was reached regarding adherence to the ICHD-3 criteria in routine clinical practice for diagnosis and classification of migraine with participants agreeing that the criteria effectively define migraine and are easy to follow. However, experts in the panel noted that ICHD-3 has certain limitations, which include a lack of specific details regarding refractory migraine (migraine non-responsive despite treatment), intensity of headache, presence of premonitory symptoms, and trigger factors. Since migraine diagnosis is dependent on patient self-report, there is a risk of underdiagnosis, as mild-intensity attacks remain unreported and the ICHD-3 definition requires a minimum of 5 attacks (20). Hence, identifying such pitfalls is essential to ensure that patients are appropriately diagnosed and offered timely preventive treatment.

The primary goal of migraine preventative treatment is to reduce the frequency and severity of attacks while addressing any comorbid conditions. Treatment selection depends on factors such as migraine type (episodic or chronic), disability level, treatment history, tolerability, comorbidities, and patient preferences (21). Management strategies are typically categorised as abortive or preventive. Abortive treatments are often associated with medication overuse and increased headache frequency, along with treatment-emergent adverse effects, often necessitating the use of prophylactic treatment (1, 22). Current preventive therapies such as calcium channel blockers, antidepressants, antiepileptic drugs, and botulinum toxin A, often show variable efficacy and tolerability, leading to suboptimal adherence and highlighting the need for alternative options (15, 23). CGRP, a potent vasodilator elevated during migraine attacks and central to pain signalling, sensitization, and allodynia, represents a compelling therapeutic target, with preventive treatments now including anti-CGRP mAbs and oral CGRP-receptor antagonists (gepants) that have demonstrated efficacy in both acute and preventive settings (24–26); however, access to these agents remains limited across many parts of Asia which made the current preventive therapies remain the mainstay of care for many patients in APAC, particularly where newer agents are unavailable or not reimbursed.

Currently, four anti-CGRP mAbs: erenumab, fremanezumab, galcanezumab, and eptinezumab are available for the treatment of migraine. Consistent with clinical trial data showing meaningful reductions in MMDs and benefits even in patients with prior preventive treatment failures, including evidence from the FOCUS trial demonstrating efficacy of fremanezumab in patients unresponsive to up to four preventive therapy classes, the expert panel in this study reached consensus that anti-CGRP mAbs effectively fulfill an unmet need for migraine prevention (27–29). However, individual mAbs differ in their safety and efficacy profiles and participants in this study placed greatest weight on efficacy, followed by safety and tolerability for selecting an appropriate anti-CGRP mAb among patients with chronic or episodic migraine. This pattern is broadly in line with previous international Delphi consensus in migraine, which have also emphasised the need for flexible, individualised preventive strategies and highlighted that preventive treatment decisions must account for patient-specific factors and evolving therapeutic options (30, 31). However, our expert panel placed relatively greater weight on cost and access, reflecting the constraints of many health systems in the Asia–Pacific region. The introduction of CGRP-targeted therapies has further prompted a re-evaluation of traditional stepwise treatment algorithms, with recent position statements and reviews advocating a more prominent role for these agents in preventive care (32, 33). Our study extends these insights to the Asia-Pacific context, highlighting how updated guidance on diagnosis and treatment is applied across diverse health-care systems in the region, where access and reimbursement constraints are often more pronounced than in relatively homogeneous national settings (34, 35).

Adolescents appear to be more susceptible to migraine than younger children, likely due to hormonal fluctuations and psychosocial changes associated with puberty. A study by Bottcher et al. found that post-pubertal girls experienced migraine more frequently than their pre-pubertal counterparts, with evidence suggesting a cyclical hormonal influence on attack frequency (36). Similarly, research by Martin et al. indicated that behavioural changes linked to puberty also contributed to increased migraine frequency in adolescents (37). Supporting this, a South Korean study reported significantly higher prevalence, frequency, and symptom severity of headaches in adolescents compared to younger children (38). In parallel, emerging evidence is beginning to clarify the pharmacokinetics and safety of anti-CGRP monoclonal antibodies in younger populations. As a result, current recommendations support the use of these agents in post-pubertal adolescents (39). Consistent with this, all participants in the present study agreed to consider anti-CGRP mAb treatment only for adolescents aged 12–17 years, unanimously excluding children aged 6–11 years. The recently published results from the Phase 3 SPACE-EM study showed that over a 3-month period, fremanezumab significantly reduced MMD compared to placebo (−2.5 vs. − 1.4; *p* = 0.0210). Additionally, in adolescents aged 12–17 years, fremanezumab led to greater improvements from baseline, with least squares mean changes in MMD of −2.7 vs. −1.8 for placebo (40). This convergence between expert opinion and the evolving evidence base underscores the importance of age-appropriate application of these therapies.

Adherence and compliance to treatment are crucial for optimal outcomes in chronic conditions and are primarily impacted by dosing frequency (41). In a study conducted by Cowan et al., patients demonstrated a slight preference for quarterly dosing, as compared to monthly dosing (42). In the present Delphi, participants’ opinions were distributed across monthly dosing, quarterly dosing, and no specific preference, with universal agreement that patient preferences should be incorporated into treatment decisions. However, access to anti-CGRP therapies in APAC remains significantly limited due to high costs and limited public or insurance coverage. Financial barriers especially in low- and middle-income countries, limit the affordability and uptake of these treatments, with high out-of-pocket costs being associated with poorer treatment adherence, which in turn leads to increased healthcare resource utilisation (43–45). In this Delphi, experts ranked cost, availability, and reimbursement above dosing regimen and “wearing-off” concerns when choosing an anti-CGRP mAb, underscoring that particularly in the APAC region, the glaring unmet need is patient access, with affordability and equitable access being key determinants of real-world impact. These findings also underline that APAC is not a homogeneous region; participating countries such as Australia, China, Japan, South Korea, Thailand, and Taiwan differ markedly in funding models, referral pathways, and availability of specialist care and advanced therapies. In addition, epidemiological and survey data from East Asia and the broader Asia–Pacific region consistently show low consultation rates and underdiagnosis of migraine, suggesting that cultural factors and health-seeking behaviour further influence how expert recommendations are implemented in routine practice (7, 46).

This study has strengths and limitations. With regards to strength, the Delphi method is recognised for reaching consensus through expert opinions in situations of uncertainty or dichotomous evidence or where formal clinical trials are particularly challenging or impractical (47–49). Additionally, high response rates suggest high engagement, interest and relevance to clinical practice, supported by 100% response to both the surveys and good attendance and participation in an online meeting. Anonymous supervised feedback and the presence of an independent moderator supported the robustness of the process.

With regards to limitations, the participants of the study are highly engaged headache specialists in migraine management who are well versed with the most recent developments in the field. Their opinions may not be reflective of community practitioners. It is unknown whether a different group of experts or experts from different countries would reach similar conclusions, thereby limiting the generalizability of these results. The results of this study have temporal validity and may change with evolving knowledge, evidence, and practises in migraine management. In addition, many of the participating experts have active collaborations and relationships with pharmaceutical companies, which may introduce potential conflicts of interest that should be considered when interpreting their consensus, particularly regarding anti-CGRP therapies. However, the Delphi questionnaire was developed and validated by an independent, non-sponsor headache specialist who served as chairperson of the study, who also moderated the scientific meeting, which helped to minimise sponsor-related influence on statements phrasing and interpretation of results.

## Conclusion

Consensus was achieved on several key aspects of migraine diagnosis and management. Participants agreed that the ICHD-3 criteria represent the preferred standard for diagnosing migraine. For assessment, they favoured the use of a headache diary as the primary tool, followed by the MIDAS and HIT-6 scales. Additionally, there was consensus that anti-CGRP monoclonal antibodies effectively address the unmet need for migraine prevention. Panel members also agreed that safety, tolerability, and efficacy are the primary factors considered, while cost and availability are also considered if the previously mentioned criteria are fulfilled. The participants agreed that dosing frequency and route of administration have an impact on treatment adherence. Finally, participants advised against the use of anti-CGRP monoclonal antibodies in children under 12 years of age due to a lack of sufficient clinical evidence. They highlighted the need for more robust data to support the use of these therapies in paediatric populations.

## Data Availability

The original contributions presented in the study are included in the article/Supplementary material, further inquiries can be directed to the corresponding authors.
