# Supplementary material for: Delphi consensus on the real-world application of the updated diagnostic and treatment guidelines for migraine in Asia Pacific
Source: Front Neurol. 2026 May 28;17:1770966. doi: 10.3389/fneur.2026.1770966 (PMC13253248; doi:10.3389/fneur.2026.1770966)
Supplement: Supplementary file 1 [file Table_1.DOCX]

Table S1: Summary of all statements evaluated during both surveys for diagnosis of migraine

| **Q. No.** | **Question** | **Survey 1** | **Survey 2** | **Overall** |
| --- | --- | --- | --- | --- |
| **Current clinical diagnosis of Migraine** | | | | |
| **Q1a** | **In your routine clinical practice, do you follow ICHD-3 diagnostic criteria for diagnosis and classification of migraine?** | **Positive (93%)** | NR | **Positive** |
| **Q1b** | **In your routine clinical practice, other than ICHD-3, do you follow any other diagnostic criteria for diagnosis and classification of migraine?** | NR | No (13) | Positive |
|  |  |  | Yes (1) | No (13) |
|  |  |  |  | Yes (1)* |
| **Q2** | **Are you aware of and do you agree with the ICHD-3 definition in migraine for the following 4 criteria?** | | | |
|  | -At least 5 or more attacks in lifetime including fulfilling the below 3 criteria | **Positive (93%)** | NR | **Positive** |
|  | -Headache attack lasting 4-72 hours | **Positive (79%)** | NR | **Positive** |
|  | -At least 2 out of 4 features (unilateral location, pulsating/throbbing quality, moderate-severe intensity, aggravation by/causing avoidance of routine physical activity) | **Positive (93%)** | NR | **Positive** |
|  | -At least 1 of the following features during headache (nausea and/or vomiting, photophobia and phonophobia) | **Positive (86%)** | NR | **Positive** |
| **Q3** | **With your clinical expertise, do you believe that the ICHD-3 diagnostic criteria are easy to follow in diagnosis and classification of migraine?** | **Positive (79%)** | NR | **Positive** |
| **Q4a** | **In your routine clinical practice, when diagnosing migraine, do you assess the following?** | | | |
|  | -Migraine disability | **Yes (100%)** | NR | **Yes** |
|  | -Depression | **Yes (100%)** | NR | **Yes** |
|  | -Anxiety | **Yes (93%)** | NR | **Yes** |
|  | -Sleep Disturbance | **Yes (86%)** | NR | **Yes** |
|  | -History of medication overuse | **Yes (100%)** | NR | **Yes** |
|  |  | Medication taking history & Headache diary (7), Interview (3), Questionnaire (2), Both Medication taking history & Headache diary and Questionnaire (1), Clinical (1) |  |  |
| **Q4b** | **If Yes to 4a, In your routine clinical practice, when diagnosing migraine, Do you use a validated assessment tool?** | | | |
|  | -Migraine disability | **Yes (12/14)** | NR | As per Survey-1 data |
|  |  | MIDAS (3), HIT-6 (1), Both MIDAS & HIT-6 (8) |  |  |
|  |  | **No (2/14)** |  |  |
|  |  | Clinical (1), Frequency of headache (1) |  |  |
|  | -Depression | **Yes (10/14)** | NR | As per Survey-1 data |
|  |  | PHQ-9 (5), HAMD-17 (2), Both PHQ-9 and HAMD-17 (1), Both PHQ9 and HAD scale (1), BDI (1) |  |  |
|  |  | **No (4/14)** |  |  |
|  |  | Clinical (1), Interview (1), Clinical history and evaluation(1), daily routine of the patient and patient's coping behavior (1) |  |  |
|  | -Anxiety | **Yes (9/13)** | NR | As per Survey-1 data |
|  |  | GAD-7 (4), HAS (2). SAS (3) |  |  |
|  |  | **No (4/13)** |  |  |
|  |  | Clinical (1), Interview (1), Clinical history and evaluation(1), daily routine of the patient and patient's coping behavior (1) |  |  |
|  | -Sleep Disturbance | **Yes (7/12)** | NR | As per Survey-1 data |
|  |  | PSQI (5), Both PSQI and ESS (1), Insomnia Severity Index (1) |  |  |
|  |  | **No (5/12)** |  |  |
|  |  | Clinical (2), Interview (1), Sleep parameters*(1), History taking (1) |  |  |
|  | -Any other criteria when diagnosing migraine | ICHD-3 (1), Visual Aura Rating Scale 2016 American College of Rheumatology Criteria for Fibromyalgia AND Restless Leg Screening Questionnaire (1), Migraine screener (1), Historical evolution of symptoms, past / current MOH, migraine associated features, Family history, comorbid conditions impacting migraine & treatment, other medications and recent changes, reproductive & menstrual history, diet, caffeine, exercise, hydration, work / hobbies / ergonomics, previous treatments / investigations (1), trigger factors - response to specific migraine acute therapy (1) | | |
| **Q5** | **Which of the following validated diagnostic assessment/screening tools do you use when evaluating the clinical findings in patients with chronic/episodic migraine? *Please rank the priority of usage considering 1=most preferred, 5=least preferred*** | | | |
|  | -Headache diary mainly looking into MMD (monthly migraine days) | **Rank 1** | NR | Rank 1 |
|  | -Quality of life assessment using MIDAS (Migraine disability assessment scale) | **Rank 2** | NR | Rank 2 |
|  | -Quality of life assessment using HIT-6 (6-Item Headache impact test) | **Rank 3** | NR | Rank 3 |
|  | -Quality of life assessment using MSQ (Migraine-Specific Quality of Life Questionnaire) | **Rank 4** | NR | Rank 4 |
|  | -Quality of life assessment using 24 h MQoLQ (24-hour Migraine Quality of Life Scale) | **Rank 5** | NR | Rank 5 |
|  | -Any other diagnostic assessment/screening tool | MIBS-4 to assess interictal burden (1), Diary also looking at acute medication use, clear days, severity and multi-day attacks (1) | | |
| **Q6** | **In your routine clinical practice, do the migraine patients present with the following?** | | | |
|  | -Bilateral headache | **Yes (14/14)** | NR | **Yes** |
|  |  | Average (43% Present Bilateral headache) |  | Average (43%) |
|  | -Unilateral headache | **Yes (14/14)** | NR | **Yes** |
|  |  | Average (57% Present Unilateral headache) |  | Average (57%) |
| **Q7a** | **Do you consider "neck pain" as a common clinical symptom of migraine?** | **Yes (13/14)** | NR | **Yes** |
| **Q7b** | **If Yes, What proportion (percent) of patients would experience neck pain?** | **<25%- 1** | NR | **<25%- 1** |
|  |  | **>25-50%- 6** |  | **>25-50%- 6** |
|  |  | **>50-75%- 6** |  | **>50-75%- 6** |
|  |  | **>75%- 1** |  | **>75%- 1** |
| **Q8a** | **Do you consider "dizziness" as a common clinical symptom of migraine?** | **Yes (13/14)** | NR | **Yes (13/14)** |
| **Q8b** | **If Yes, What proportion (percent) of patients would experience dizziness?** | **<25%- 7** | NR | **<25%- 7** |
|  |  | **>25-50%- 5** |  | **>25-50%- 5** |
|  |  | **>50-75%- 1** |  | **>50-75%- 1** |
| BDI - Beck Depression Inventory, ESS - Epworth Sleepiness Scale, GAD-7 - Generalized Anxiety Disorder 7-item scale, HAD scale - Hospital Anxiety and Depression scale, HAMD-17 - Hamilton Depression Rating Scale (17 items), HAS - Hamilton Anxiety Scale, HIT-6 - Headache Impact Test, ICHD-3 - International Classification of Headache Disorders (3rd edition), MIDAS - Migraine Disability Assessment, MMD - Monthly Migraine Days, MOH - Medication Overuse Headache, MQoLQ - Migraine Quality of Life Questionnaire, MSQ - Migraine-Specific Quality of Life Questionnaire, PHQ-9 - Patient Health Questionnaire-9, PSQI - Pittsburgh Sleep Quality Index, SAS - Self-Rating Anxiety Scale | | | | |

Table S2: Summary of all statements evaluated during both surveys - treatment of migraine

| **Q. No.** | **Question** | **Survey 1** | **Survey 2** | **Overall** |
| --- | --- | --- | --- | --- |
| **Updated treatment concepts with anti-CGRP mAbs in Migraine preventive management** | | | | |
| **Q9** | **Do you agree that monoclonal antibodies (mAbs) targeting the CGRP pathway are effective and fulfill the unmet need in patients with chronic/episodic migraine?** | **Positive (86%)** | NR | **Positive** |
| **Q10** | **What would be the deciding factor in choosing a specific anti-CGRP mAb for patients with chronic/episodic migraine?** | | | |
|  | -Efficacy | **Positive (100%)** | NR | **Positive** |
|  | -Safety | **Positive (93%)** | NR | **Positive** |
|  | -Tolerability | **Positive (86%)** | NR | **Positive** |
|  | -Frequency of administration | **43%** | Updated the question in survey-2 as below | |
|  | -Wearing off effect | 43% |  |  |
|  | -Availability/Reimbursement | 57% |  |  |
|  | -Cost (if it is an out-of-pocket expenditure for the patient) | 58% |  |  |
| **Q10b** | **In addition to efficacy, safety and tolerability, how would you rank accordingly the importance of the following deciding factors in choosing a specific anti-CGRP mAb for patients with chronic/episodic migraine? *Please rank most preferred=1 and least preferred=4*** | | | |
|  | -Cost (if it is an out-of-pocket expenditure for the patient) | Updated the question in survey-2 | Rank 1 | Rank 1 |
|  | -Availability/Reimbursement |  | Rank 2 | Rank 2 |
|  | -Frequency of administration |  | Rank 3 | Rank 3 |
|  | -Wearing off effect |  | Rank 4 | Rank 4 |
| **Q11** | **If cost is not an issue, in which line of treatment do you think the anti-CGRP mAbs should be advised to patients with chronic/episodic migraine?** | First line- 11/14 | NR | 1^st^ line-79% |
|  |  | Second line- 3/14 |  | 2^nd^ line-21% |
| **Q12** | **If migraine patients have responded to anti-CGRP mAbs in the follow-up visit/assessment, how long do you consider to further treat the patient with anti-CGRP mAbs?** | 6 months- 5/14 | NR | 6 months- 36% |
|  |  | 12 months- 6/14 |  | 12 months- 43% |
|  |  | >12 months- 3/14 |  | >12 months- 21% |
| **Q13a** | **When prescribing anti-CGRP mAbs to your patients, do you have any preference in the dosing regimen?** | Monthly regimen- 4/14 | NR | Monthly regimen- 28% |
|  |  | Quarterly regimen- 5/14 |  | Quarterly regimen- 36% |
|  |  | No preference- 5/14 |  | No preference- 36% |
| **Q13b** | **Would you also take into consideration the patients’ preference in this case?** | **Yes (14/14)** | **NR** | **Yes** |
| **Q14a** | **Do you treat migraine in children/ adolescents (6-17 years old) in your routine clinical practice?** | **Yes (10/14)** | **NR** | **Yes** |
| **Q14b** | **If Yes, for pediatric use, in which age group would you consider treating patients with anti-CGRP mAbs?** | 6-12 years (0/10) | **NR** | 13-17 years |
|  |  | 13-17 years (10/10) |  |  |
| **Q15a** | **Do you believe that** **there is an unmet need of combining with or switching to anti-CGRP mAbs while patients with chronic/episodic migraine are already taking other migraine preventive agents?** | | | |
|  | ***Updated the question in survey-2 as:*** | | | |
|  | **When patients with chronic/episodic migraine have failed (no response or partial response) oral migraine preventive agents*, do you agree to prescribe (either combine with or switch to) advanced therapies like anti-CGRP mAbs?** | | | |
|  | -Topiramate | **7%** | **Positive (93%)** |  |
|  | -Divalproex sodium/valproate sodium | **14%** |  |  |
|  | -Beta-blocker: e.g. metoprolol, propranolol, timolol, atenolol, nadolol | **7%** |  |  |
|  | -Tricyclic antidepressant: e.g. amitriptyline, nortriptyline | **14%** |  |  |
|  | Serotonin-norepinephrine reuptake inhibitor: e.g. venlafaxine, duloxetine | **21%** |  |  |
|  | -You prefer combining or switching any other preventive agents with anti-CGRP mAbs, please specify? | Combination with Gepants (1), I am not sure what response is expected in Q15a - do you mean a medication-specific challenge in combing with or switching to anti-CGRP mAb by "unmet need"? (1), Candesartan and onabotulinum toxin A, for possible mechanistic synergy (1) | | |
| **Q15b** | **If you agree to combining with or switching to anti-CGRP mAbs, what do you prefer?** | | | |
|  | ***Updated the question in survey-2 as:*** | | | |
|  | **If you agree to combining with or switching to anti-CGRP mAbs, what do you prefer? Please select** | | | |
|  | Option1: Switching to anti-CGRP mAbs when there is no response with other migraine preventive agents | 7/8 | 10/14 | 71% |
|  | Option2: Combining with anti-CGRP mAbs when there is a partial response with other migraine preventive agents | 8/8 | 10/14 | 71% |
|  | Option3: Combining with anti-CGRP mAbs when there is no response with other migraine preventive agents | NR | 5/14 | 36% |
|  | Option4: Switching to anti-CGRP mAbs when there is partial response with other migraine preventive agents | NR | 5/14 | 36% |
| **Q16a** | **Recently published RCT data has shown positive clinical outcomes in patients with chronic/episodic migraine with comorbidity such as “depression”, how likely would it be that anti-CGRP mAbs is prescribed as a treatment option in such patients with “major depressive disorder (MDD)”?** | **Positive (79%)** | NR | **Positive** |
| **Q16b** | **Do you prefer referring such patients to a psychiatrist and vice versa?** | Yes (10/14) | NR | Yes (72%) |
|  |  | No (4/14) |  |  |
|  | If yes,, do you prefer referring such patients to a psychiatrist and vice versa? | | | |
|  | -I usually get referrals from a psychiatrist | 0/10 | NR | 0% |
|  | -I usually refer such patients to a psychiatrist | 3/10 | NR | 30% |
|  | -Both of the above options | 7/10 | NR | 70% |
| **Q17** | **In terms of safety, adherence, and compliance, which preventive treatment would have a better clinical outcome in patients with chronic/episodic migraine?** | | | |
|  | ***Updated the question in survey-2 as:*** | | | |
|  | **In terms of safety, adherence and compliance, which preventive treatment taken alone, in your clinical opinion, would have a better clinical outcome in patients with chronic/episodic migraine?** | | | |
|  | -Anti CGRP mAbs | 5/14 | 13/14 | 93% |
|  | -Gepants | 1/14 | 1/14 | 7% |
|  | -Both | 8/14 | NR |  |
| **Q18** | **Would you agree that while prescribing anti-CGRP mAbs/Gepants, dosing regimen and route of administration has an impact on the patient’s adherence to the treatment?** | **Positive (86%)** | NR | **Positive** |
| CGRP - Calcitonin Gene-Related Peptide, MDD - Major Depressive Disorder, mAbs - Monoclonal Antibodies, RCT - Randomized Controlled Trial | | | | |
